# Disparate effects of ankle-brachial index on mortality in the ‘very old’ and ‘younger old’ populations-the PolSenior survey

**DOI:** 10.1007/s00380-021-01949-1

**Published:** 2021-10-13

**Authors:** Jarosław Królczyk, Anna Skalska, Karolina Piotrowicz, Małgorzata Mossakowska, Tomasz Grodzicki, Jerzy Gąsowski

**Affiliations:** 1 Department of Internal Medicine and Gerontology, Jagiellonian University Medical College, Krakow, Poland; 2 University Hospital in Krakow, Krakow, Poland; 3 International Institute of Molecular and Cell Biology in Warsaw, Warsaw, Poland

**Keywords:** Ankle-brachial index, Mortality, Older age, PolSenior study

## Abstract

To assess the relationship between ankle-brachial index (ABI) and up to 10-year mortality in older individuals below and above the age of 80 years. In a multicenter survey of health status in the community dwelling subjects aged 55–59 and 65 + years in Poland, we assessed baseline medical history including risk-factors. We measured ABI, and serum creatinine, cholesterol, NT-proBNP, and interleukin-6 (IL-6) concentrations. We assessed mortality based on public registry. Between 2009 and 2019, 27.3% of 561 participants < 80 years, and 79.4% of 291 participants ≥ 80 years, died (*p < *0.001); 67.8, 41.5, and 40.3% in the ABI groups < 0.9, 0.9–1.4, and > 1.4, respectively (*p < *0.01). In the unadjusted Cox models, ABI was associated with mortality in the entire group, and < 80 years. In the entire group, analysis adjusted for age and sex showed mortality risk increased by 11% per year, and 50% with male sex. Mortality decreased by 37% per 1 unit ABI increase. In the group of people ≥ 80 years, only age was significantly associated with mortality (*p < *0.001). In stepwise regression ABI < 0.9, male sex, active smoking, and NT-proBNP level were associated with risk of death < 80 years. In the ≥ 80 years old, mortality risk was associated with older age, and higher levels of IL-6, but not ABI. The ABI < 0.9 is associated with higher mortality in older people, but not among the oldest-old. In the oldest age group, age is the strongest predictor of death. In this age group, inflammageing is of importance.

## Introduction

### Background

Ankle-brachial index (ABI) 0.9 has been used to diagnose peripheral arterial disease (PAD), even at the pre-clinical stage [1]. Low ABI is associated with atherosclerosis of coronary and carotid arteries [2–6], and increased all-cause and cardiovascular mortality [5–19]. ABI of more than 1.4, an index of arterial stiffness, has also been associated with adverse outcome, especially in older subjects [8, 20, 21]. Age is a potent modifier of risk, and passing the age of 80 years is considered to be a threshold beyond which relation between risk-factors and outcome may change. Few studies, yielding inconclusive results addressed the issue of ABI in relation to hard outcome in the very old persons [22, 23].

The aim of this study was to assess the relationship between baseline ABI, in the context of other cardiovascular risk factors, and the mortality of participants of the PolSenior survey in older individuals below and above the age of 80 years.

## Methods

### Study population

The PolSenior study was a nationwide, multicentre, cross-sectional survey of health status and its determinants in the community dwelling subjects aged 55–59 and 65 and more years [24]. Research participants were randomly recruited from among the population of Poland, in a stratified, proportional draw performed in three stages. In the first stage, the municipalities were selected according to the population, in the second the street addresses, and in the case of rural communities the villages, and in the third stage, based on the Ministry of Internal Affairs and Administration personal identification data the individuals in bundles were selected [24]. Between January 2009 and April 2010, we enrolled 5695 participants. In 1018 (17.8%), medical examination was performed by geriatricians. In the current report, we present the data from 852 participants in whom we obtained the data on ABI on the right lower limb, and for whom vital status was available as of 20th April 2019.

### Data collection

Sociodemographic data, information about habits, and past medical history were obtained using a structured questionnaire administered by trained nurses during home visits. As part of the geriatric assessment, basic activities of daily living (ADL) and the Tinetti gait and balance test were performed, with higher values associated with better preservation of function [25, 26].

Medical examination by the geriatrician included ABI measurement according to the guidelines. Blood pressure was measured on both arms with an A&D, UA 787 Plus automatic device, in supine position, after 5-min rest. In case of between-arm difference of blood pressure, higher values were used for subsequent analyses.

The systolic blood pressure (SBP) on both posterior tibial arteries was assessed using Doppler Bidop ES-100VX device with 8 MHz ultrasound probe (Hadeco, Inc, Kawasaki, Japan). Blood pressure cuff was positioned on a calf with the distal edge of the cuff 3 cm above the medial malleolus. The SBP measurement was performed on both lower extremities. ABI was calculated as: systolic blood pressure on posterior tibial artery (mm Hg)/systolic blood pressure on brachial artery (mm Hg) [27]. There were five missing values for the left lower extremity, and the average difference between right and left lower extremity values was not statistically significant (*p* = 0.27). Accordingly, we decided to use the values from right lower extremity in all subsequent analyses.

For the stratified analyses, we categorized ABI into normal: 0.9–1.4, low: < 0.9, and high: > 1.4. Body mass index (BMI) was calculated according to the formula: body weight (kg)/height (m^2^). Creatinine and cholesterol levels were measured by the standard method with an automated analyzer Modular PPE (Roche Diagnostics) and Roche Diagnostics GmbH reagents (Mannheim Germany), and the LDL cholesterol was measured directly with enzymatic-colorimetric method.

A serum aminoterminal pro-brain natriuretic peptide  (NT-proBNP) level was measured by an electrochemiluminescence immunoassay (ECLIA) method (Roche Diagnostics GmbH, Mannheim, Germany) with a immunoassay analyzer Cobas e411 (Roche Diagnostics GmbH, Mannheim, Germany).

Plasma interleukin 6 (IL-6) concentration was determined by high-sensitivity ELISA using R&D Systems kits (Minneapolis, MN, USA).

Mortality was assessed 10 years after beginning of the study. Data on deaths were obtained from the personal id number (PESEL) register, after obtaining the consent of the Ministry of Digitization. The study was approved by the Bioethics Committee of the Medical University of Silesia in Katowice (KNW-6501–38/I//08). Each participant gave written, informed consent to take part in the study.

### Statistical analysis

The database management and the statistical analyses were performed with SAS 9.4 (SAS Institute Inc., Cary, NC, USA). Continuous variables were presented as means with standard deviations, and compared with the standard normal *Z*-test, in case of normal distribution, and as medians with interquartile ranges and compared with Wilcoxon’s test. The proportions were compared with Chi square test. To demonstrate the influence of ABI at baseline on 10-year mortality, we first plotted the Kaplan-Maier curves, for the entire group and in individuals under and over 80, where the ABI status was categorized into 3 subcategories (< 0.9, 0.9–1.4, > 1.4). Where applicable, the analyses were adjusted for multiple comparisons with Bonferroni method. Further, we used Cox regression to model time to event (death) as a function of baseline ABI. The models were fitted for entire group and in the subgroups under 80 and over 80 years of age. Each time we started with unadjusted model, then fitted a model with adjustment for sex and age and finally on top of ABI, sex, and age, the stepwise procedure selected significant confounders form among the following: SBP, DBP, IL-6, LDL cholesterol, NT-proBNP, creatinine, BMI, current smoking status; and the history of each of the following: myocardial infarction (MI), stroke, heart failure, diabetes mellitus, and hypertension. In the survival analyses, cases with no event and follow-up shorter than 10.4 years were right-censored.

## Results

The characteristics of study population divided into groups according to the age is shown in Table 1. Low ABI was present in 118 (13.9%), normal ABI in 662 (77.7%) and high ABI in 72 (8.4%) participants, respectively.

**Table 1:** Characteristics of participants depending on age, in groups below 80 years of age and aged 80 and over

Variable	< 80 years of age *N* = 561[mean (Std); median (interquartile range); %]	≥ 80 year of age *N* = 291 [mean (Std); median (interquartile range); %]	*p*
Age (years)	68.6 (7.2)	86.3 (4.5)	< 0.001
Sex (% men)	50.1	58.8	0.048
BMI (kg/m^2^)	28.9 (5.06)	27.1 (4.53)	< 0.001
Active smokers	15.0	5.1	< 0.001
ABI	1.13 (0.23)	1.04 (0.30)	< 0.001
SBP (mmHg)	141.4 (19.9)	143.7 (23.2)	00.23
DBP (mmHg)	80.3 (11.2)	75.79 (11.6)	< 0.001
Hypertension (%)	69.5	71.6	0.59
Myocardial infarction (%)	10.2	14.2	0.11
Heart failure	5.9	11.7	0.003
Stroke (%)	4.3	11.0	< 0.001
Diabetes mellitus (%)	22.3	21.9	0.85
Creatinine (mg/dl)	0.888 (0.21)	1.032 (0.36)	< 0.001
LDL (mg/dl)	122.8 (40.1)	112.3 (36.3)	< 0.001
NT-proBNP (ng/l)	0.135 (0.07–0.26)	0.32 (0.19–0.73)	< 0.001
IL-6 (pg/ml)	1.7 (1.1 – 2.7)	2.6 (1.8–4.3)	< 0.001
Tinetti (sum)	25.8 (5.1)	19.7 (8.1)	< 0.001
ADL (sum)	5.9 (0.5)	5.4 (1.2)	< 0.001
Survival time (years)	8.4 (2.2)	5.6 (2.8)	< 0.001

*BMI* body mass index, *ABI* ankle-brachial index, *SBP* systolic blood pressure, *DBP* diastolic blood pressure, *LDL* low-density cholesterol

The time between entry into the study and assessment of vital status averaged 9.4 years (range 8.4 to 10.5, years) years. During the average 9.4-year follow-up, 153 (27.3%), and 231 (79.4%) participants died in the group of < 80 years of age, and in the group ≥ 80 years (*p < *0.001), respectively. In the entire age-range, the mortality was: 67.8% (80 persons) in the low ABI group, 41.5% (275 persons) in the normal ABI group and 40.3% (29 persons) in the high ABI group, respectively (*p < *0.001).

**Fig. 1:**
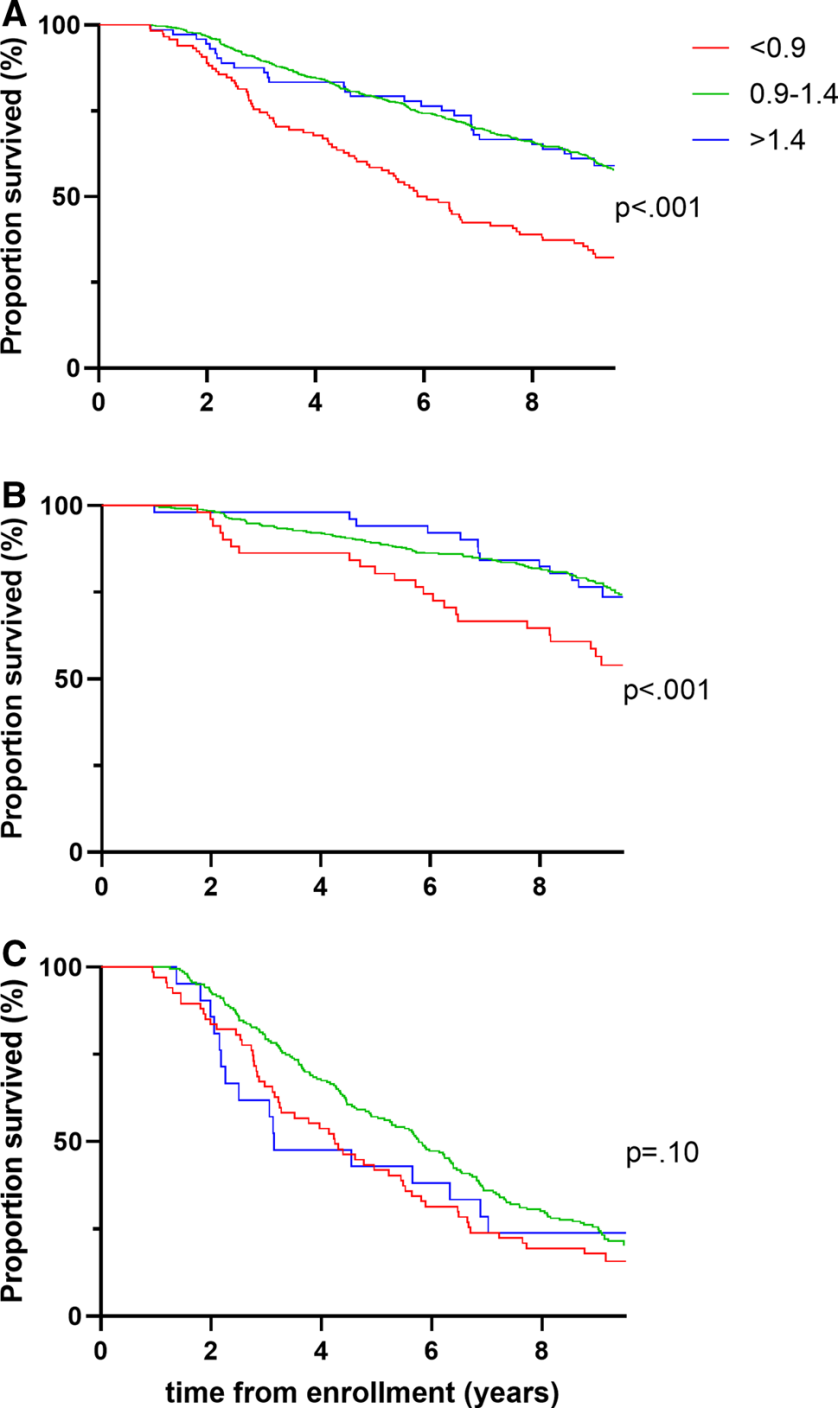
Survival according to ankle-brachial index status in the entire group (**A**) and in groups < 80 (**B**) and ≥ 80 (**C**) years of age

Kaplan–Meier survival analysis in the entire group revealed significantly higher mortality rate in low ABI group in comparison to people with ABI within normal range and with high ABI (Log-rank test, *p < *0.001) (Fig. 1). The mortality did not differ between the two latter groups (Fig. 1).

Similar results were obtained in the subgroup of participants below 80 years of age. Mortality was significantly higher in individuals with ABI < 0.9 in comparison to persons with normal and high ABI (Log-rank test, *p < *0.001). Mortality did not differ between normal range ABI and ABI > 1.4 (Fig. 1).

In the group ≥ 80 years, the mortality rate over a 9.4-year period did not differ according to the ABI status (*p* = 0.10) (Fig. 1).

In the unadjusted models, ABI was associated with mortality in the entire group and in subgrou*p < *80 years of age, but not in oldest subgroup. In the entire group, analysis adjusted for age and sex showed that mortality risk increased by 11% for each year of life, by 50% for male sex, and decreased by 37% for 1 unit greater ABI (Table 2). Similar direction of relationship was demonstrated in persons under 80 years of age, however in men, the risk of death was more than two-fold higher than in women, and 1 unit higher ABI value was associated with mortality risk lower by 62%. In similar analyses in the group ≥ 80 years, only age was significantly associated with mortality (*p < *0.001). In this age group, neither sex nor ABI were associated with a risk of death (Table 2).

**Table 2:** Relative hazard ratios for mortality estimated with ankle brachial index lower by 1 unit-Cox regression results. Elypsis denotes that the variable did not enter or stay in the model (*p* > 0.05)

Model	All *n* = 852 RHR (95% CI)	*p*	< 80 years *n* = 561 RHR (95% CI)	*p*	≥ 80 years *n* = 291 RHR (95% CI)	*p*
Unadjusted						
ABI	0.33 (0.22 – 0.50)	< 0.001	0.42 (0.21 – 0.85)	0.016	0.70 (0.43 – 1.14)	0.15
Adjusted for age and sex						
Age	1.11 (1.10 – 1.12)	< 0.001	1.12 (1.08 – 1.15)	< 0.001	1.12 (1.09 – 1.15)	< 0.001
Male sex	1.50 (1.22 – 1.84)	< 0.001	2.26 (1.62 – 3.16)	< 0.001	1.14 (0.88 – 1.49)	0.33
ABI	0.63 (0.43 – 0.93)	0.019	0.38 (0.20 – 0.73)	0.004	0.84 (0.53 – 1.34)	0.47
Stepwise adjusted for potential confounders with age, sex, and ABI forced into the model						
Age	1.11 (1.09 – 1.12)	< 0.001	1.12 (1.08 – 1.15)	< 0.001	1.13 (1.10 – 1.17)	< 0.001
Male sex	1.31 (1.03 – 1.67)	0.027	2.32 (1.60 – 3.36)	< 0.001	0.90 (0.67 – 1.21)	0.50
ABI	0.95 (0.62 – 1.48)	0.83	0.45 (0.23 – 0.90)	0.02	0.86 (0.52 – 1.42)	0.56
BMI	0.97 (0.95 – 1.00)	0.047	–	–	–	–
Diastolic blood pressure	1.01 (1.00 – 1.02)	0.03	–	–	–	–
Smoking	1.46 (1.01 – 2.12)	0.046	2.24 (1.40 – 3.58)	< 0.001	–	–
NT-proBNP	1.20 (1.09 – 1.33)	< 0.001	1.82 (1.37 – 2.41)	< 0.001	–	–
IL-6	1.03 (1.01 – 1.05)	0.002	–	–	1.03 (1.01 – 1.06)	0.007
Stepwise adjusted for potential confounders with age, sex, and ABI forced into the model, additionally adjusted for ADL						
Age	1.11 (1.10 – 1.12)	< 0.001	1.11 (1.07 – 1.14)	< 0.001	1.13 (1.10 – 1.17)	< 0.001
Male sex	1.38 (1.09 – 1.76)	0.008	2.35 (1.62 – 3.40)	< 0.001	0.92 (0.68 – 1.23)	0.56
ABI	0.94 (0.60 – 1.46)	0.77	0.49 (0.25 – 0.98)	0.04	0.86 (0.52 – 1.42)	0.55
Diastolic blood pressure	1.01 (1.00 – 1.02)	0.049	–	–	–	–
NT-proBNP	1.19 (1.09 – 1.31)	< 0.001	1.86 (1.40 – 2.48)	< 0.001	–	–
Smoking	1.56 (1.08 – 2.25)	0.02	1.97 (1.23 – 3.16)	0.005	–	–
ADL	0.84 (0.75 – 0.95)	0.004	0.58 (0.42 – 0.80)	0.001	–	–
IL-6	–	–	–	–	1.03 (1.01 – 1.06)	0.007
Stepwise adjusted for potential confounders with age, sex, and ABI forced into the model, additionally adjusted for Tinetti test						
Age	1.09 (1.08 – 1.11)	< 0.001	1.11 (1.07 – 1.15)	< 0.001	1.11 (1.07 – 1.14)	< 0.001
Male sex	1.48 (1.17 – 1.87)	0.001	2.59 (1.78 – 3.76)	< 0.001	1.08 (0.80 – 1.46)	0.63
ABI	0.86 (0.56 – 1.31)	0.47	0.42 (0.21 – 0.85)	0.02	1.06 (0.64 – 1.78)	0.81
NT-proBNP	1.20 (1.09 – 1.31)	< 0.001	1.62 (1.19 – 2.20)	0.002	1.16 (1.04 – 1.30)	0.008
Smoking	1.67 (1.16 – 2.41)	0.006	2.43 (1.51 – 3.92)	< 0.001	–	–
Tinetti	0.95 (0.93 – 0.96)	< 0.001	0.94 (0.91 – 0.97)	< 0.001	0.95 (0.93 – 0.97)	< 0.001
LDL	–	–	–	–	1.004 (1.001 – 1.008)	0.03

Variables offered into the model, that did not enter or stay for any subgroup: systolic blood pressure, LDL, creatinine, hypertension, diabetes mellitus, history of myocardial infarction, history of heart failure, history of stroke

In the final step, after forcing sex, age, and ABI into the model, the stepwise procedure was employed to select important confouders from among the following baseline characteristics: smoking status, BMI, systolic and diastolic blood pressure, serum levels of creatinine, IL-6, LDL, and NT-proBNP, presence of hypertension, diabetes, heart failure, history of stroke and myocardial infarction. In these analyses, ABI was associated with mortality only in the subgroup aged < 80 years. ABI higher by 1 unit decreased mortality risk by 55%. In the entire group, increased risk of death was associated with older age, male sex, lower BMI, higher diastolic blood pressure, higher NT-proBNP and IL-6 concentration and active smoking (Table 2).

Low ABI value, next to age, male sex, active smoking, and increased NT-proBNP level was significantly associated with risk of death in the group aged < 80 years (Table 2).

According to the results of stepwise regression performed in the group aged ≥ 80 years, mortality risk was associated with older age, and higher levels of IL-6, but not with ABI (Table 2). These results were not materially altered when ADL (Table 2) or results of Tinetti test were added to stepwise procedure.

## Discussion

We demonstrated that ABI of < 0.9 is associated with increased mortality in older persons, as opposed to both normal (0.9–1.4) and increased (> 1.4) ABI values. This was driven by the results in participants below the age of 80 years. In the octogenarian-and-older group, ABI status did not influence mortality.

We based our results on a 9.4-year follow-up for vital status, of a large subsample from a nation-wide study of health of older persons and its determinants that was carried out in Poland. We demonstrated mortality gradient from low ABI (67.8%), to normal (41.5%) and high ABI (40.3%). Previously, the estimated two to fourfold increase of risk of mortality [14] associated with low ABI was confirmed in a number of populations. These included high-risk patients diagnosed with cardiovascular (CVD) disease [18], burdened with CVD risks factors [11], symptomatic and asymptomatic PAD [9, 12, 17], and diabetes mellitus [13, 15, 18]. The risk was demonstrated in the primary care setting [10, 13], and in population-based studies [4, 5, 8, 16–19], alike. This was further confirmed in systematic reviews and meta-analyses [16, 28, 29]. Two studies published thus far assessed 10-year mortality according to ABI status. Mueller et al. [15] showed three-fold higher risk of death associated with low ABI in comparison to the matched controls, but study was carried out in the symptomatic PAD group with a mean age of 64 years, which may correspond to our younger group aged < 80 years. Sartipy et al. [17] obtained similar results in a population-based observational study of 4940 subjects with a mean age of 71 years. They showed that the 10-year all-cause mortality was 27% in the control group, 56% in the asymptomatic PAD, 63% in the intermittent claudication, and 75% in the severe limb ischaemia groups, respectively.

The incidence of low ABI increases with age reaching 23% to 60% after the age of 802 [30, 31]. Previously, we showed that in the Polish older population abnormal ABI was found in 31.8%; the value of ABI < 0.9 in 19.9%, and ABI > 1.4 in 11.9% of examined persons [32].

Few studies focused on the relationship between the ABI value and the risk of death in people over 80 years of age. Murabito et al. [22] in a 4-year follow-up of 251 men and 423 women with mean age of 80 years and Bo et al. [23] in study group of 632 nursing home residents with mean age of 82 years showed that ABI < 0.9 was not related to all-cause and cardiovascular mortality. Our results extend this evidence into over twice as long a follow-up and onto community dwelling very old subjects.

In our study, the characteristics of the groups < 80 years and ≥ 80 years, are similar, however, the older subgroup was characterized by slightly greater percentage of men, lower frequency of smoking, two times higher prevalence of heart failure and higher median NT-pro BNP level, higher frequency of stroke, higher IL-6 concentration, lower DBP and lower LDL level. No significant differences were observed between the study groups in terms of diabetes mellitus, BMI, hypertension, and myocardial infarction. We did not find relation between medical history and risk of mortality.

These results, and in particular the lack of significant effect of diabetes on mortality, contradicts the results of other studies [10, 12, 15, 18]. Our Cox models with stepwise selection of significant explanatory variables demonstrated that in the < 80 years group, in addition to low ABI, survival was shortened by older age, male sex, smoking and elevated NT-proBNP level. In the ≥ 80 years old group, only age and IL-6 levels had a significant impact on survival. These results are consistent with other studies [10, 12]. In the German Get ABI study during 3-year follow-up of persons with mean age of 72 years, in addition to ABI < 0.9, the older age, male sex, smoking, diabetes, and CVD episodes have been associated with mortality, while hyperlipidemia and hypertension were not significantly associated with the fatal outcome [10]. In turn, in the study by Mueller et al. [12] which included data from the Linz Peripheral Arterial Disease study, the factor affecting mortality was the elevated NT-proBNP concentration, and additionally, in diabetic patients, a higher hsCRP level.

The observation that in our group above the age of 80 years classic cardiovascular risk factor did not influence outcome may reflect the fact that at the advanced age other factors, such low grade inflammation, may play more important role. The higher concentration of IL-6 shown in our group of subjects ≥ 80 years of age, and its significant negative impact on survival are in line with other studies conducted in the populations of older persons [33]. Aging is associated with an increasing activation of the entire inflammatory cascade resulting in systemic low-grade inflammation. The inflammaging, is defined as 2 – fourfold increase in inflammation-related cytokines, acute phase proteins and elevated count of inflammatory cells [34]. Moreover, it is recognized that local inflammatory processes and systemic low-grade inflammation were characteristic parts of the pathology in almost all chronic, age-associated diseases. The association of chronic low grade inflammation with risk of death was demonstrated in a number of studies conducted in the population of people older than 80 years, showing the association between markers of inflammation and the cardiovascular mortality independently of common cardiovascular risk factors such as sex, hypertension, hypercholesterolemia, BMI, low physical activity [34–36].

In none of the analyses did we find a relationship between high ABI, a marker of medial arterial calcification and arterial stiffness, and mortality. The mortality did not differ between ABI of 0.9–1.4 and > 1.4. The results presented in the literature are contradictory. Some reports documented increased mortality in high ABI population [8, 20, 21], while others showed no relationship similar to our results [4, 16, 19, 37–39].

Our results need to be interpreted in the context of their possible limitations. First, our follow-up for mortality, which was based on public registry, did not include the cause of death. Likewise we did not perform assessment of non-fatal events, including cardiovascular events and hospitalisations. On the other hand, our data are based on a planned follow-up, and the subjects were drawn from a sample representative at the national level. The subsample we used was prespecified in the protocol of our survey to undergo technical examination.

In conclusion, the ABI below 0.9 is associated with higher mortality in older people, but not among the oldest-old, over 80 years of age. In the oldest age group, age and inflammation seem to be the strongest risk factors for all-cause mortality. Although, surprisingly, the ADL was not predictive of mortality in the oldest group, the Tinetti test result was. Thus, our results underpin the need for employment of the comprehensive geriatric assessment in all older patients, largely irrespective of their cardiovascular status.

## Data Availability

Available upon reasonable request.

## Code availability

SAS Base codes.
